# Health Tract No. 338

**Published:** 1868-07

**Authors:** 


### HEALTH TRACT NO. 338.

### MORAL ANNOYANCES.

Many a man has fallen dead on the reception of bad news; the other day a thoughtless youth galloped up to a house and told the lady that her husband had just been killed, she was thrown into convulsions and died in a few moments; impressions less disagreeable affect the health proportionably; sometimes persons are found to be distressingly sensitive to particular occurrences; some there are who are almost ready to go into a fit on observing a person to eat with his knife, or to pick his teeth at table with the prong of a fork, or spitting tobacco juice on a nice carpet, especially if the carpet belongs to the observer. There are situations in which one person may inflict grievous annoyances on a dozen, a hundred, even thousands, of persons at the same time; smoking in church for example, coming into church or lecture or concert or readings after the commencement of the exercises, a proof of ill breeding and vulgarity equal to a demonstration; singing in church, out of time or so loud as to drown all surrounding voices; striking want of tidiness in dress or toilet. These moral annoyances and health disturbers are met with when in reading our favorite newspaper, sly digs are given the clergy, the Sabbath day, the Bible, the Chirstian religion and woman; or when reading a magazine to observe every now and then that it steps out of its way to insult or to expend its wit upon a particular religious sect or class of society or political party; it is an unmitigated meanness to take advantage of position, in these directions. It is a growing annoyance among the clergy to hear them, in the exposition of the Scriptures, use such expressions as these, " or more properly translated;" "a better rendering would be;" " the sense would have been more strikingly conveyed if read " so and so. If a minister cannot manage to preach from the Bible as it is, he had better try his hand at something else; all the attempts to improve the Bible impair the confidence of the masses in its infallibility as a guide; and it would be greatly better to select some text for proof of a particular position, which could be made to bear upon it without any alteration whatever; besides, the very assumption of superior scholarship implied, is exceedingly offensive and out of place. " Don't let any Doctor persuade you to take any medicine for your complaint," said a Professor in the College of Physicans and Surgeons of New York City to a gentleman, a few days ago." " A great many sins are charged to the liver now a days by the doctors; its a great mistake," said this same person in our hearing recently, in a lecture " to young men only." With " great applause," said the papers next morning. Said applause was proportioned to the smuttiness of the remarks in every case. A statement of the immediate cause of each of the " cheers," would have been suggestive. A medical editor in his May number says, "Doctors have begun to exercise common sense, and have ceased to blindly follow blind guides." We think that he who attempts to elevate himself by the depression of his own class, calling, or profession, lacks the true ring of a noble nature.

